# Anti-Coagulant and Antimicrobial Recombinant Heparin-Binding Major Ampullate Spidroin 2 (MaSp2) Silk Protein

**DOI:** 10.3390/bioengineering9020046

**Published:** 2022-01-19

**Authors:** Pranothi Mulinti, Dorina Diekjürgen, Kristen Kurtzeborn, Narayanaganesh Balasubramanian, Shane J. Stafslien, David W. Grainger, Amanda E. Brooks

**Affiliations:** 1 Department of Pharmaceutical Sciences, North Dakota State University, Fargo, ND 58105, USA; pranothimulinti@gmail.com; 2 Department of Pharmaceutics and Pharmaceutical Chemistry, University of Utah, Salt Lake City, UT 84132, USA; Dorina.diekjuergen@utah.edu (D.D.); kurtzebornk@gmail.com (K.K.); david.grainger@utah.edu (D.W.G.); 3 Proteomics, Metabolomics and Mass Spectrometry, North Dakota State University, Fargo, ND 58105, USA; b.narayanaganesh@gmail.com; 4 Coatings and Polymeric Materials, North Dakota State University, Fargo, ND 58105, USA; shane.stafslien@ndsu.edu; 5 Department of Biomedical Engineering, University of Utah, Salt Lake City, UT 84112, USA; 6 Department of Molecular Biology, Rocky Vista University, Ivins, UT 84738, USA

**Keywords:** spider silk, heparin binding peptides, recombinant protein expression, functional heparin assay, fusion protein

## Abstract

Governed by established structure–property relationships, peptide motifs comprising major ampullate spider silk confer a balance of strength and extensibility. Other biologically inspired small peptide motifs correlated to specific functionalities can be combined within these units to create designer silk materials with new hybrid properties. In this study, a small basic peptide, (ARKKAAKA) known to both bind heparin and mimic an antimicrobial peptide, was genetically linked to a protease-resistant, mechanically robust silk-like peptide, MaSp2. Purified fusion proteins (four silk domains and four heparin-binding peptide repeats) were expressed in *E. coli*. Successful fusion of a MaSp2 spider silk peptide with the heparin-binding motif was shown using a variety of analytical assays. The ability of the fusion peptide to bind heparin was assessed with ELISA and was further tested for its anticoagulant property using aPTT assay. Its intrinsic property to inhibit bacterial growth was evaluated using zone of inhibition and crystal violet (CV) assays. Using this strategy, we were able to link the two types of genetic motifs to create a designer silk-like protein with improved hemocompatibility and antimicrobial properties.

## 1. Introduction

**Figure 1:**
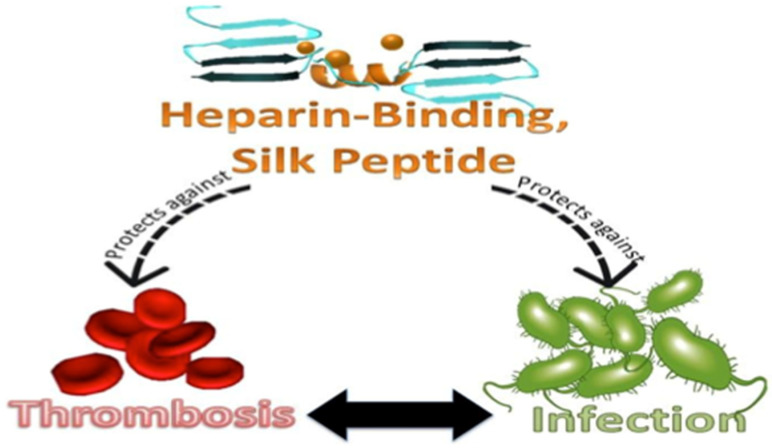
Infection and thrombosis are intimately connected. This is of particular concern for hemodialysis catheters and other blood-contacting medical devices. Using the naturally occurring heparin-binding motif (HBM) with the silk peptide can both bind heparin to alter coagulation and provide antimicrobial properties, ultimately improving blood compatibility and limiting infection risks.

A hierarchical structure rooted in the biochemical composition of two main proteins, MaSp1 and MaSp2, gives major ampullate spider silk an impressive balance of strength and elasticity, unrivaled by synthetic materials [1,2]. Phylogenetic analyses of major ampullate silk sequences, in addition to other proteomic [3] and structural analyses [4,5], revealed evolutionarily conserved repetitive amino acid motifs subsequently correlated with specific structural and functional features of the silk [6]. These correlative conclusions have been well-studied by producing clones of simplified monomer motifs [4,7,8,9] to identify and establish overarching structure–property relationships [6,10]. In addition to mechanical properties that can be manipulated by genetically combining functional motifs, several recombinant spider silk fusion proteins have been explored to date (i.e., silk–uranium binding proteins [11], silk–antibiotic [12], silk–silica binding proteins [13,14], silk–bone sialoprotein [15], silk–elastin [16] etc.). Alternatively, spider silk protein was also recently chemically complexed with heparin and chitosan to create an anticoagulant, antimicrobial, dual-function protein [17]. This manuscript describes a new genetic modification of silk designed to both capture circulating heparin and provide intrinsic antimicrobial properties (Figure 1).

Heparin, a hydrophilic, highly sulfonated, negatively charged polysaccharide copolymer comprised of glucosamine and glucuronic acid, is endogenously found in the human body (approximately 20 µg/mL in adult plasma). It is also commonly used as an extracorporeal anticoagulant at therapeutic concentrations over 50 nM [18,19]. Importantly, heparin is naturally bound to a diverse family of heparin-binding proteins based largely on electrostatic interactions [20,21,22]. Positively charged basic amino acid residues in the consensus Heparin Binding amino acid Motif (HBM) of these proteins interacts through ionic and hydrogen bonds with the strong negatively charged sulfate and carboxylate groups on the heparin polysaccharide [20,23]. Furthermore, the tertiary structure of the HBM is analogous to the overall structure (XBBBXXBX, where X is a hydrophobic or uncharged amino acid and B is a basic amino acid) of an antimicrobial peptide (AMP) [24,25]. Consequently, previous work has sought to exploit surface-bound HBM to create heparinized surfaces [26,27]. These efforts were able to successfully bind solution-phase heparin, with the best binding affinity based on four concatenated repeats of the synthetic motif (ARKKAAKA, K_D_ = 42 ± 15 nm) [27]. Importantly, this general peptide sequence is analogous to the LL-37 antimicrobial peptide, which is sensitive to proteolysis by human neutrophil-produced elastase as well as *P. aeruginosa* elastase, *S. aureus* V8 metalloproteinase and aureolysin [28]. Accordingly, bound heparin bioactivity was shown to be reduced by proteolytic cleavage [26,29]; therefore, utilizing a heparin-binding motif as a surface capture agent for circulating heparin necessitates embedding the protease-susceptible peptide into a protective polymer background. A biological and structural biopolymer, such as major ampullate spider silk, exhibiting no similar protease sensitivities may provide a suitable background to afford stability and permit heparin capture. In this context, we sought to genetically embed a small heparin-binding amino acid motif (HBM) into a recombinant silk-like protein [30,31,32,33], ultimately creating a genetic chimera with new biomaterial properties (conceptually described in Figure 1).

Although many other biologically active silk fusion proteins have been explored [34] (e.g., antibiotics [12,35], chitosan [17], elastin [16], silica [13,14,36], RGD [37]), the combination reported here is a unique embodiment. Four concatenated repeats of the heparin-binding motif, ARKKAAKA, were genetically linked with MaSp2 amino acid motifs (GPGXXA_n_) to produce a mechanically robust protein capable of both (1) binding biologically active heparin and (2) resisting pathogen colonization was produced, potentially enabling both in vivo anticoagulation and anti-infectivity [38]. The anti-coagulant activity of this fusion protein was comparable to the activity of surface-bound heparin. Here we report the design, production, and characterization of a new recombinant silk fusion protein with the ability to bind heparin and resist bacterial adhesion and growth.

## 2. Materials and Methods

### Protein Expression and Purification

The complete genetic sequence of either two repeats of silk (SX2) or four repeats of silk linked with four repeats of HBM (S4H4) were designed, synthesized by GenScript (Piscataway, NJ, USA), and cloned into pET 30a for expression (Table 1).

All proteins were expressed in *E. coli* BL21(DE3) pLysS cells (Promega, Fitchburg, MA, USA). Cultures were grown by shaking (220 rpm) at 37 °C in Luria broth (LB) containing 100 µg/mL kanamycin to an OD_600_ between 0.8 and 1.0. Isopropyl-β-D-1-thiogalactopyranoside (IPTG, Sigma-Aldrich, St. Louis, MI, USA) was added at a final concentration of 1 mM to induce protein expression. After 2 h, the culture was centrifuged at 8000 rpm for 10 min, and the media was decanted. Cell pellets were resuspended in 100 mM HEPES buffer (1/10 of the final culture volume). To achieve better yields and higher purity, 50 µg/mL DNase I (Sigma-Aldrich) and 1μL of protease inhibitor cocktail (Sigma-Aldrich) was added per 100 mL of harvested culture prior to sonication (30 s at 40% power) and purification. Proteins were purified using nickel affinity chromatography. To identify the highest yield clones from each construct, the Maxwell^®^ 16 Polyhistidine Protein Purification system (Promega) was used according to the manufacturer’s instructions for the prefilled nickel affinity reagent cartridge (Promega) [39]. Purified protein was dialyzed against water overnight and lyophilized [40].

## 3. Protein Characterization

### 3.1. Sodium Dodecyl Sulfate Polyacrylamide Gel Electrophoresis (SDS-PAGE)

Purified proteins were analyzed for size and purity by SDS-PAGE. Solution from each well of the Maxwell purification cartridge was analyzed to assess the efficacy of the purification. Each sample was briefly (<5 min) heated with NuPAGE LDS sample buffer (Invitrogen, Waltham, MI, USA) at 95 °C and then loaded into a precast Bis-tris 4–12% Polyacrylamide gradient gel (Invitrogen). The gel was run in MES buffer at 150 V for 45 min. Protein bands were visualized on a BioRad Chemidoc XRS imaging system after staining with AcquaStain Protein Gel Stain (Bulldog Bio. Inc, Portsmouth, NH, USA).

### 3.2. Western Blot

The identity of purified protein products was confirmed via western blotting. Purified proteins were run on a denaturing SDS-PAGE and transferred to a nitrocellulose membrane at 40 V for 60 min using a standard transfer protocol for semi dry blotting [41]. After proteins were transferred to the nitrocellulose membrane using Invitrogen’s Xcell™ Blot Module, the SNAPi.d. protein detection system (Milliporesigma, St. Louis, MI, USA) was used for immune detection with a 1:4000 dilution of a HRP-conjugated 6x-His Epitope Tag Polyclonal Antibody (PA1-23024, ThermoFisher, Waltham, MI, USA) in Tris buffered saline plus Tween 20 (0.05%) (Fisher, USA TBST, ionic strength = 175 mM). Amersham™ ECL™ Prime Western blotting detection reagent (GE Healthcare Life Sciences, Chicago, IL, USA) was used according to manufacturer specifications, and the bands were visualized using the Bio-Rad ChemiDoc™, Hercules, USA XRS camera under varying exposure.

### 3.3. Mass Spectrometry (MS)

Mass spectrometry was performed at the Core Synthesis and Analytical Services Facility (Center for Protease Research, North Dakota State University, Fargo, ND, USA) on purified protein samples. Briefly, the intact mass analyses of the specific silk-like proteins was performed after desalting them followed by LC-MS analysis on a Waters Synapt G2-Si HDMS (Waters Corporation, Milford, CT, USA). UPLC was performed on an Acquity UPLC-I class with Waters BEH C18 (2.1 mm × 100 mm) 1.7 µm column. The column was maintained at 35 °C throughout the analyses. A linear gradient was performed over 7 min, shifting the ratio of A (0.1% formic acid in water) to B (0.1% formic acid in acetonitrile) from 90/10 (A/B) to 10/90 (A/B). The total gradient run was 13 min. Desalted protein solution (100 µL) was mixed with 200 µL of 0.1% TFA in water/acetonitrile (50/50) and 10 µL was injected at a rate of 0.5 mL/min. Mass spectrometric analysis was performed on a Waters Synapt G2-Si HDMS. The collected mass spectrum data in continuum format were processed using MaxEnt1 software (Waters Corporation) to obtain the protein mass. The peak width parameter used to obtain the result was between 0.45 Da to 0.6 Da depending on the sample. Spectra were processed between 5000 to 50,000 Da at 1 Da/channel.

## 4. Heparin-Binding Characterization

### 4.1. Heparin Affinity Dot Blot

Purified proteins (10 µL at approximately 100 µg/µL) were applied to a nitrocellulose membrane (Sigma-Aldrich) and allowed to adsorb for 30 min. The membrane was blocked on a rotating platform for 1 h in a blocking solution (5% non-fat instant milk) (Carnation, Los Angeles, CA, USA) in Tris buffered saline plus Tween 20 (0.05%) (TBST, Fisher, USA) (ionic strength = 175 mM). The membrane was subsequently probed with biotinylated heparin (Sigma-Aldrich; from porcine intestinal mucosa, MW 15,000 Da, purity: >97%) diluted at 1:100 in the blocking solution for 1 h on a rotating platform. After washing the membrane three times for 15 min each in TBST (approximately 75 mL), the blot was probed with horseradish peroxidase (HRP)-tagged streptavidin (Fisher Scientific, Hampton, NH, USA) diluted 1:100 in the blocking solution for 1 h on a rotating platform. The membrane was washed again three times for 15 min each in TBST (approximately 75 mL), and reacted with enhanced chemiluminescence reagents (Pierce, Rockford, IL, USA) for 5 min prior to imaging on an Aplegen Omega Lum G, San Francisco, USA over 5 min, acquiring images at varying exposure times. An equivalent amount of Interleukin-2 (IL-2, PeproTech, East Windsor, NJ, USA), documented to bind heparin [42], was used as positive control while BSA, with no documented ability to bind heparin, was used as the negative control for all assays [43,44]. IL-2 binding of heparin did not yield a positive result in ELISA (data not shown). Additionally, a separate blot was probed with secondary streptavidin–HRP (Horseradish Peroxidase) (ThermoFisher Scientific, Waltham, MA, USA) only using an analogous protocol without the addition of biotinylated heparin.

### 4.2. Heparin Affinity ELISA

An indirect ELISA was performed on protein samples with BSA and plain silk (SX2) as negative controls. Polystyrene microtiter plate wells (n = 3) were incubated with 100 µL of protein samples at a concentration of 100 μg/mL in Tris buffer (pH 7.5) for 2 h. After incubation, the wells were washed three times using TBST (0.05% Tween) and blocked using 5% milk in TBS for another hour. Plates were rinsed again with TBST three times. Subsequently, 100 µL of biotinylated heparin (heparin-biotin sodium salt, MW 15,000 Da, Sigma Aldrich, St. Louis, MO, USA) at a concentration of 45 µg/mL diluted in TBST was added to each well and incubated for 1 h. Wells were washed again with TBST three times before adding streptavidin conjugated with HRP at a dilution of 1:1000 as the secondary ligand and incubated for 1 h. Wells were again washed, and 100 μL of TMB substrate (Fisher Scientific) was added and allowed to incubate for 20 min at which time the reaction was stopped using 1 M HCl, according to the manufacturer’s instructions for the TMB substrate. Optical absorbance was read at 480 nm on an Epoch plate reader (Biotek, Winooski, VT, USA).

### 4.3. APTT Coagulation Assay

An activated partial thromboplastin time (aPTT) assay, which assesses the rate at which the complex of plasma clotting factors assembles to convert prothrombin to thrombin prior to clot formation, (APTT XL Pacific Homeostasis Assay, ThermoFisher,) was performed to indirectly assess blood coagulation in the presence of silk binding motifs SX2 and S4H4 according to the manufacturer’s protocol. Whole blood for the assay was collected from euthanized rats via cardiac puncture into a sodium citrate (4% *w*/*v*) solution and immediately stored at −80 °C for use. Blood was thawed and then centrifuged at 2000 rpm for 10 min to separate the plasma. Initially, heparin was titrated into plasma to determine the amount needed to prevent citrate-treated plasma from clotting in the presence of calcium. Briefly, aqueous heparin (heparin sodium porcine mucosa, Sigma Aldrich) (200 µL per well), ranging from 50 μg/mL to 200 μg/mL (diluted in Tris buffered saline (TBS)) was incubated with plasma (0.1 mL) in each well (n = 2) for 2 h at room temperature with shaking. Subsequently, 0.1 mL of APTT-XL reagent was added, and the reaction was incubated for 5 min. Calcium chloride solution (0.1 mL of 0.02 M) was added to initiate clot-like formation. The time required for the formation of the clot was recorded. Clot-like formation was visually assessed as an optical change in the transparency of the solution. Based on its ability to prevent clot formation during the titration, 100 μg/mL of heparin was used for all additional assessments. A similar protocol was followed to assess the coagulation of plasma in the presence of SX2 and S4H4 with the exception that the wells (n = 4) were incubated with either SX2 or S4H4 proteins at a concentration of 200 μg/mL prior to the addition of heparin, plasma or both. After allowing the protein to attach to the surface for 2 h, the protein-depleted solution was removed from the wells and the wells were rinsed with PBS three to five times and blocked with 0.5% milk in TBS for 30 min. The wells were not allowed to completely dry. Subsequently, all wells were rinsed again three times with PBS, and 200 μL of heparin (100 μg/mL in TBS) was added to the wells and incubated for another 2 h. After incubation, the wells were rinsed with PBS and activated thromboplastin time was assessed using the APTT-XL assay by adding clarified plasma (0.1 mL) and the APTT-XL reagent (0.1 mL). Silk and S4H4 without heparin were used as controls.

## 5. Antibacterial Activity

### 5.1. Kirby Bauer Zone of Inhibition Assay

Maxwell-purified S4H4 and SX2 protein solutions (300 μL of 20 mg/mL, ~90% purity) were spotted on the surface of an LB agar plate (within 15 min) previously streaked with *E. coli* (ATCC 12435). An equivalent amount of BSA was applied as a negative control. Only one protein was applied per agar plate with each protein spotted on 3 independent plates. The plates were incubated at 37 °C overnight and the diameter of the zone of inhibition (ZOI) was measured using digital calipers.

### 5.2. Biofilm Formation

Biofilm formation was characterized for *E. coli* (ATCC 12435) using a multi-well plate methodology described previously [45,46]. Prior to bacterial inoculation, 0.5 mL of protein solution (1 mg/mL) was added to four wells of a 24-well tissue culture polystyrene (TCPS) plate and incubated for 1 h. The protein-depleted solution was removed and the wells were rinsed three times with 1.0 mL of PBS. Subsequently, an overnight culture of *E. coli* prepared in LB broth was rinsed three times in PBS, harvested via centrifugation, and diluted in PBS to an OD_600_ of 0.4. The resulting suspension was used to prepare dilutions (10^7^–10^8^ cells/mL) in minimal media, M63 (10g of (NH_4_)_2_SO_4,_ 68 g of KH_2_PO_4_, 2.5 mg of FeSO_4_·7H_2_O, 254 mg of MgSO_4_·7H_2_O, pH-7) supplemented with 2 g/L dextrose. Bacterial suspensions (1 mL) were added to well plates pretreated for 1 h with either S4H4 or SX2 protein solutions and incubated statically for 24 h at 37 °C (PBS and blank plate were used as controls). The wells were rinsed three times with 1.0 mL of PBS and stained with 0.5 mL of crystal violet (CV) solution dye (Bioworld, Dublin, OH, USA) for 15 min at ambient laboratory conditions. Excess CV dye was removed by rinsing three times with 1.0 mL of water and the wells were dried for 1 h. The CV dye was extracted in 33% glacial acetic acid for 15 min to solubilize the dye bound to the biofilm. An aliquot of 150 µL of the acetic acid extracts were transferred to a 96-well plate and optical absorbance was measured at 600 nm using a multi-well plate spectrophotometer (Tecan Safire 2, Tecan US Inc., Morrisville, NC, USA).

## 6. Mechanical Testing of Silk Fibers

After recombinant proteins were desalted via dialysis and subsequently lyophilized, they were resuspended in 100 μL of hexafluoroisopropanol (HFIP) to create a viscous solution. The solution was manually extruded through a 22 G needle into isopropanol to create fibers. The diameter and birefringence of the fibers were assessed under polarized light on a Nikon DMi8 microscope using a 10× objective at 5 places along each fiber’s length. Images were analyzed using grey-scale image analysis in ImageJ (NIH). Diameter measurements were then averaged and reported with its standard deviation. Each fiber was mounted on a paper frame with cyanoacrylate glue and tensile tested (Instron 5962, Epsilon Tech, Jackson, WY, USA) at 2 mm/min per a previously published method [41].

## 7. Statistical Analysis

Data are presented as mean ± standard deviation (SD). Statistical interpretation was performed by using IBM SPSS. Statistically significant differences between the experimental groups were determined by using the Mann Whitney U test.

## 8. Results

**Figure 2:**
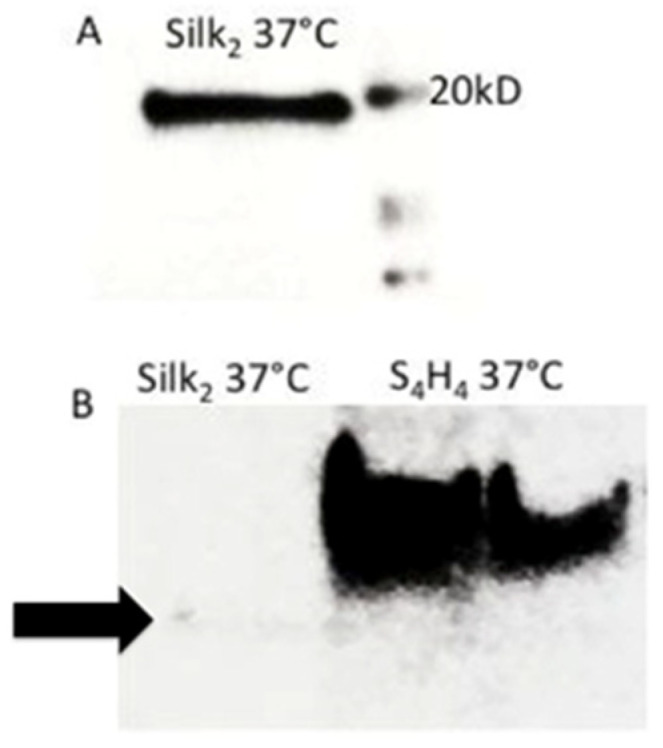
Western blots for (**A**) SX2 protein and (**B**) S4H4 (2 clones) using a HRP-conjugated anti-His antibody. Molecular weight markers are shown on SX2. Note that all gels were non-reducing.

The repetitive sequence of *Argiope aurantia* major ampullate spidroin 2 (MaSp2), one of the key structural spider silk proteins, was genetically linked with the consensus sequence for the heparin-binding motif (HBM) (Table 1 and Figure 2). MaSp2 (silk) and HBM sequences were designed and engineered not only to optimize expression in *E. coli*, but also to facilitate their genetic linkage and polymerization. All produced clones were screened via colony PCR and sequence confirmed by the University of Utah core sequencing facility (data not shown).

**Figure 3:**
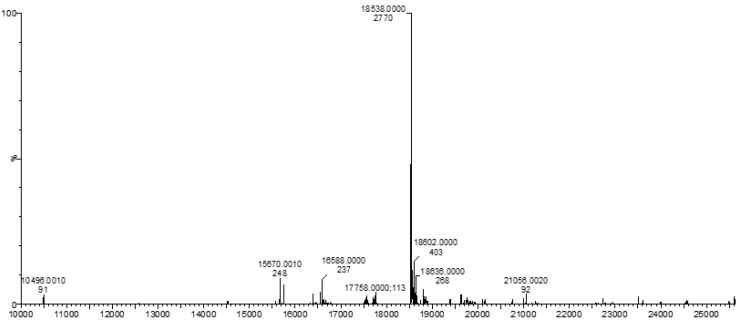
Mass spectrometric validation of the desired recombinant modified silk fusion protein sequence. Based on the amino acid sequence shown for S4H4 (Table 1), predicted to be 18,538.00 Da, the S4H4 protein was confirmed as the predominant peak in the plot.

Subsequently, genetic constructs were PCR-cloned into pET30a for protein expression. Expression was induced in BL21-DE3 *E. coli* using IPTG. An average of 10 mg protein was purified from 1 L of culture. Fusion protein production was confirmed by: (1) SDS-PAGE; (2) Western blot (Figure 2); and (3) mass spectrometry (Figure 3).

**Figure 4:**
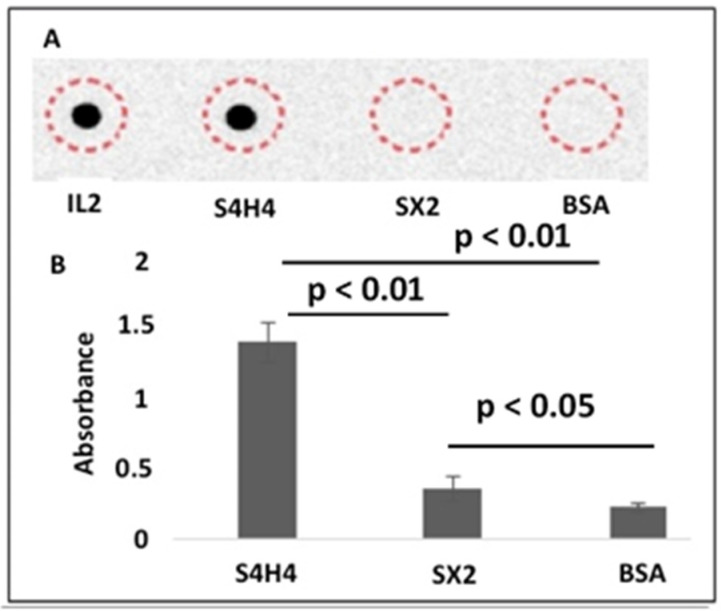
(**A**) S4H4 spotted on nitrocellulose binds heparin similar to the positive control IL-2. SX2 was unable to bind heparin, similar to the BSA negative control. A dashed circle indicates areas where each protein was spotted. (**B**) ELISA to detect the interaction of S4H4, SX2 and BSA (negative control) with heparin; n = 4, *p* < 0.05.

Following characterization of the HBM silk fusion peptide (Figure 2 and Figure 3), functional assays were used to determine its capability to bind heparin when surface-bound (Figure 4). A dot blot assay was used to confirm the ability of surface-adsorbed, fusion protein to bind biotinylated heparin (Figure 4A). The S4H4 fusion protein was detected by biotinylated heparin, whereas SX2 and BSA (negative control) were not detected, indicating that heparin was unable to bind to either protein. Notably, Bovine Serum Albumin (BSA) has no reported ability to bind heparin [45]. An analogous blot was probed with secondary streptavidin–HRP only and did not show any reaction regardless of the protein spotted (data not shown). Unfortunately, this assay format exhibited limited sensitivity: approximately 0.3 mg of protein applied to each 10 µL spot was required for detection with biotinylated heparin. Thus, an Enzyme Linked Immunosorbent Assay (ELISA) was conducted to validate the dot blot and probe the binding sensitivity. The ELISA was able to confirm that biotinylated heparin bound approximately 3-fold better to S4H4 than to SX2 or BSA (Figure 4B).

**Figure 5:**
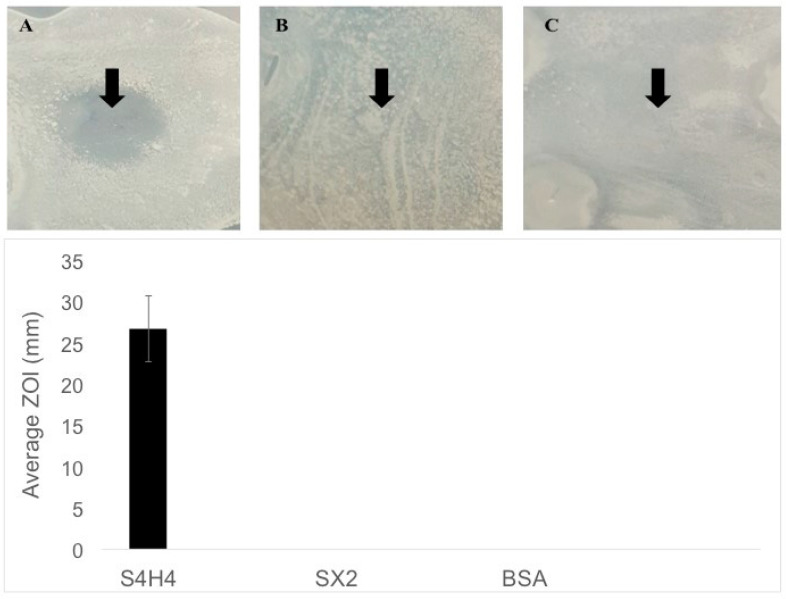
Zone of inhibition of fusion protein compared with other controls. (**A**) S4H4, (**B**) SX2, and (**C**) BSA (included as a negative control). Note that the same amount of protein was spotted (indicated by the arrow) for each sample. The average (n = 3) measured zone of inhibition is shown in the bar graph below the representative images. No zone of inhibition was observed for either silk or BSA controls.

One powerful aspect of this unique fusion is the ability of the heparin-binding motif to not only bind heparin but also to act as an antimicrobial peptide. To assess the ability of S4H4 to inhibit bacterial growth, a standard Kirby–Bauer zone of inhibition assay was performed against planktonic *E. coli* ATCC 12435. Silk without the heparin-binding motif (SX2) did not show a zone of clearing nor did BSA; however, S4H4 produced a clear zone of inhibition (Figure 5). None of the proteins complexed with heparin in this experiment.

**Figure 6:**
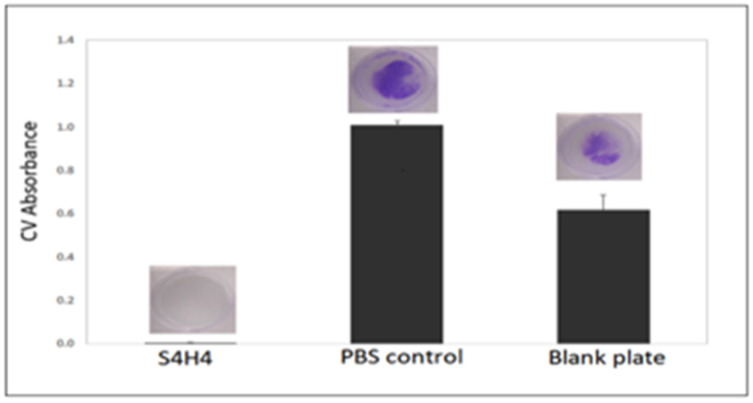
Crystal violet assay for biofilm formation showing that wells coated with S4H4 do not support biofilm formation, as evidenced by the lack of purple color. Alternatively, wells rinsed with PBS or the blank clearly evidenced biofilm (as indicated by optical density at 600 nm and presence of purple color). (n = 3).

In addition to the antibacterial effect on planktonic *E. coli* 12435, the fusion protein also prevented the growth of biofilm-like surface-adherent *E. coli* colonies (Figure 6) when compared to both PBS-treated and blank wells (i.e., TCPS) controls. ATCC *E. coli* strain 12435 is a validated biofilm-forming strain [47]. The control groups show a clear formation of biofilm with the presence of a glycocalyx as indicated by crystal violet (CV) staining (blue color) (Figure 6), whereas CV optical absorbance (600 nm), a standard measure of biofilm formation [48], in the presence of S4H4 is below the detectable limit of the assay and the spectrophotometer (L.O.D. 0.04 OD). Hence, S4H4 seems to have effectively prevented biofilm formation in the absence of heparin.

**Figure 7:**
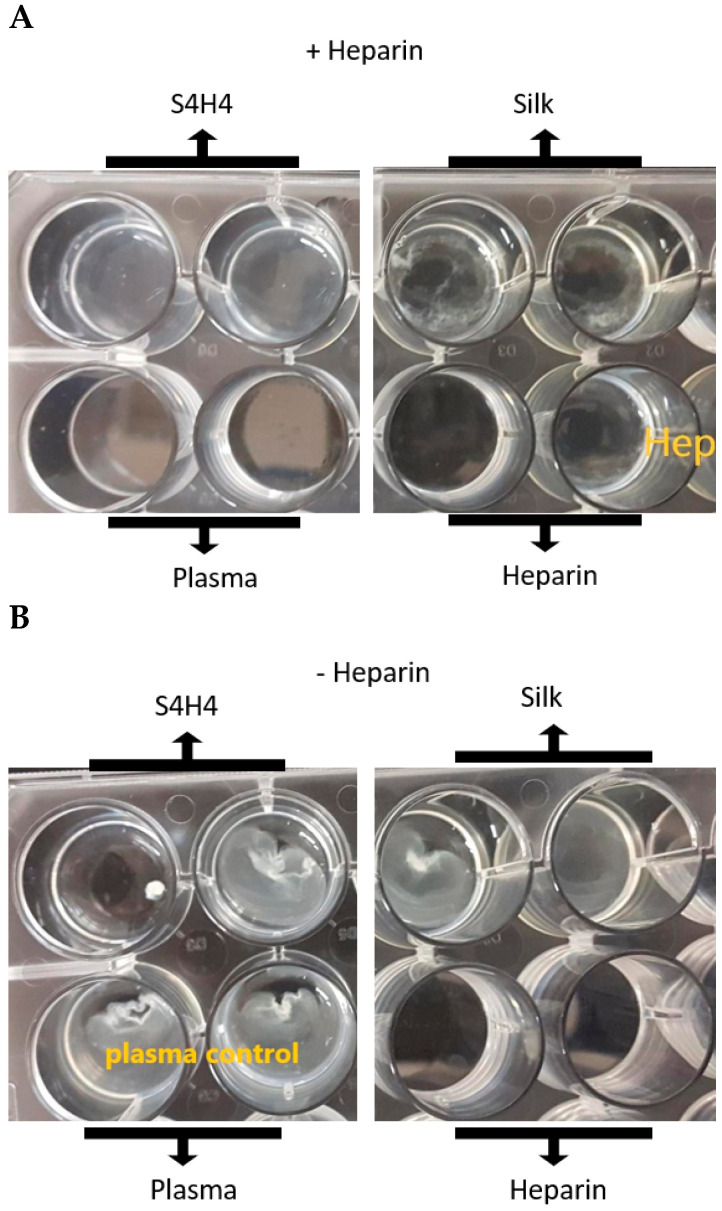
A visual representation of the aPTT coagulation assay endpoints. (**A**) heparin added to the assay produced noticeable lack of clotting in both S4H4 (top left) and the plasma control (bottom left) as anticipated by their ability to bind heparin, comparable to the heparinized controls (bottom right). The silk-coated surface shows evidence of clot-like formation (top right). (**B**) All wells lacking heparin produced a noted strong clotting response, in contrast to control heparinized wells (bottom right) (n = 3).

While both dot blot and ELISA provide evidence that heparin can bind to S4H4, bioactivity of the silk-bound heparin was also assessed. A validated assay to determine the activated partial thromboplastin time (aPTT) as an indirect measure of clot-like formation in vitro, was performed in the presence of SX2 and S4H4 proteins. Time to visualize clot formation elicited by the calcium ion addition was confirmed as a decrease in the optical transparency of the aPTT solution reaction and formation of a clot (Figure 7). When S4H4 protein was surface coated and exposed to heparinized plasma, no clot was detected by the aPTT assay after 24 h, similar to an uncoated microwell surface when soluble heparin only was added. However, when heparin was added to wells coated with SX2, clotting was merely delayed (13 ± 5 min) when compared to SX2 control wells without heparin addition (9 ± 3 min) (Table 2). Regardless of the surface coating, in the absence of heparin, SX2, S4H4 and plasma all showed similar clotting times of approximately 9 min.

**Figure 8:**
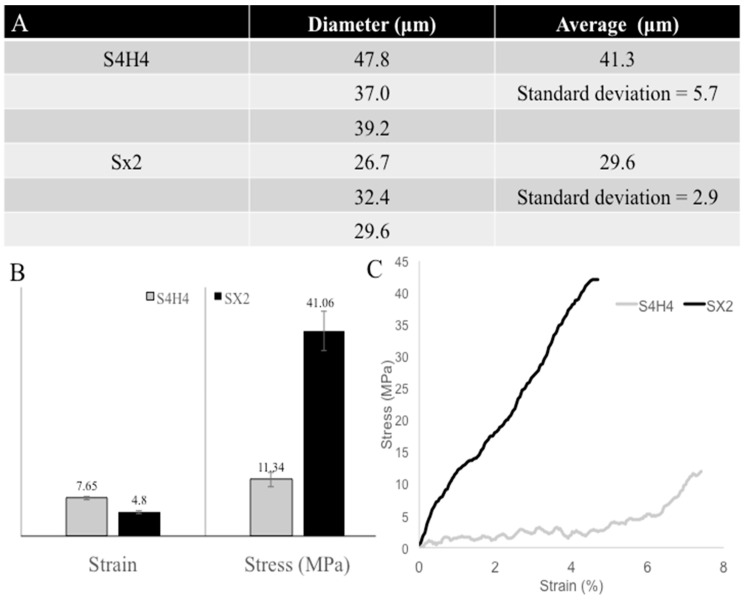
Comparison of the (**A**) wet spun fiber diameters, (**B**) average stresses and strains at breakage produced by SX2 and S4H4 fibers, and (**C**) an average stress/strain curve. S4H4 fibers were thick with decreased stress and increased strain compared to SX2.

Embedding the heparin-binding motif in a silk-like peptide was meant to endow a mechanically-robust structural peptide with the ability to bind heparin and prevent bacterial colonization of a surface. Although circular dichroism (CD, data not shown) of the fusion protein showed the presence of beta sheets and random coils, the percentage was lower than the natural silk, which could be due to the shorter length of the recombinant protein. Thus, to compare the physical properties of the recombinant proteins, fibers were wet spun from both the fusion protein (S4H4) and plain silk (SX2). Subsequent mechanical testing revealed that the presence of HBM in the fusion protein decreased the breaking stress from 41 MPa to 11 MPa, but increased the breaking strain from 4.4% to 7.1% (Figure 8). This may be a reflection of the fiber thickness, as it is known that thicker diameter fibers impart extensibility while thinner diameters are associated with strength [49]. Nevertheless, optimizing the ratio of HBM to silk may be necessary to preserve mechanical integrity.

## 9. Discussion

The relative success of surface coatings in providing a versatile platform for pharmaceutical immobilization is a driving force behind the use of either biomimetic [50,51,52] or synthetic polymers [53,54] as chemically and biologically defined surface coatings [55]. While silk’s robust mechanics and limited protease sensitivity have made it an attractive surface coating, its biochemistry and structural hierarchy have made it an accessible platform for chemical and biological modifications. Furthermore, as opposed to many surface coatings (both synthetic and biological) that provoke a robust foreign body response, the well-documented biocompatibility of silk [56] adds to its allure, particularly for blood-contacting biomaterial applications. Previously, several groups have explored heparin–silk complexes by chemically linking heparin to silkworm silk (a more readily available yet mechanically inferior variety of silk) or via blending or mixing prior to processing by electrospinning or film casting [57,58,59]. Heparin grafted either electrostatically or covalently [60] to silk-based vascular scaffolds showed that heparin grafted at a density of 1.48 ± 0.19 mg/cm^2^ was able to improve the in vitro anticoagulant effect of the silk for up to 12-weeks [61]. Despite these promising results, heparin chemically grafted to a silk surface may suffer the same fate in vivo as most other heparinized surfaces: rapid silencing of bioactivity by host protein biofouling. In the current study, the natural binding partner of heparin was genetically linked to a spider silk motif (Table 1, Figure 2 and Figure 3, Supplementary Figure S1). It was clear from the data that the S4H4 fusion protein was capable of binding heparin from a solution (Figure 4). We hypothesize that by using heparin’s binding partner instead of a chemical linkage to heparin itself, a “self-renewing”, heparin-enriched surface coating can be created. This new strategy represents a novel method that relies on the biochemistry of receptor–ligand interactions to shunt the coagulation cascade [59,60].

The theoretical “self-renewing” nature of a surface-coating produced using the reported fusion protein S4H4, which is driven by the kinetics of heparin binding, addresses one of the biggest hurdles to the efficacy of heparinized surfaces: bioactivity silencing due to protein biofouling. Although a full evaluation of the precise kinetics and stability of binding are beyond the scope of this initial in vitro study, it was noted that by varying the wash time following heparin adsorption in the aPTT assay, the ability of heparin to prevent clot formation was altered; more rigorous washing (three times for 1 min each) led to a loss of anti-coagulant activity presumably due to the loss of heparin. This supposition was confirmed via x-ray photoelectron spectroscopy (XPS, Supplementary Table S1), which showed an increase in nitrogen content and decrease in silicon after the addition of the protein, consistent with the adherent protein adsorbed layer, and a loss of protein after rigorous washing by re-appearance of these XPS protein-relevant signals. The kinetics of binding are expected to be very different in in vitro assessments of our S4H4 fusion based on the ionic strength of the assay solutions and may offer little overall relevance to the translatability of the fusion in vivo in the presence of endogenous heparins. Irrespective of the kinetics, the ability to bind heparin was not evident under any conditions considered with adsorbed SX2 or BSA negative controls (Figure 4B). Expression of the HBM peptide alone in the absence of the MaSp2 silk peptide as a positive control was not feasible (data not shown) in any measurable quantity. This is likely due to the high molar percent of lysine in the peptide and the specific bacteria’s innate ability to respond to this AMP [62,63]. Thus, the HBM peptide was expressed only as a fusion stabilized by silk.

Independent of techniques applied to determine protein ligand affinity and stability, the ionic strength of the various buffers should be considered in both binding and functional assays. Based on the nature of heparin’s molecular interactions, the ionic environment of the S4H4 fusion peptide could potentially reduce or disrupt the predominant electrostatic interactions [64] between negatively charged heparin and its positively charged protein binding partners. With over 20,000 different non-specific blood proteins and a 320 mOsm solute concentration, the substantial ionic strength of blood seems to control the specificity of natural heparin interactions [65,66]. This may indicate that a level of specificity governs the interaction of heparin beyond mere electrostatic interactions; a theory that was recently explored in the literature [67] and peripherally considered in this study. Additionally, ionic interactions may not only play a role in heparin binding and consequently anti-coagulation bioactivity, but may also influence the antimicrobial activity of the HBM. In our currently study, ELISAs for heparin–silk motif complexation were run with both desalted and buffered (Maxwell elution buffer, 500 mM imidazole, 100 mM HEPES, pH 7.5) samples with no significant differences in assay performance (data not shown), indicating that in the current study, the range of ionic strengths based on buffer composition did not alter heparin binding to HBM in S4H4 or presumably hinder interaction with SX2 or BSA controls.

Not only does incorporation of the heparin binding motif into a MaSp2 silk background provide the potential for a “self-renewing” heparin-enriched surface, but it also affords the dual functionality of being anti-coagulating and simultaneously anti-microbial. In an effort to characterize the anti-coagulant nature of the S4H4-HBM fusion protein, activated partial thromboplastin time (aPTT) assay, providing an indirect measure of coagulation (Figure 7 and Table 2), showed that rat plasma coagulation times were altered. Using this assay, the S4H4 fusion protein was asserted to bind heparin to provide functional equivalence to heparin alone, indicating that the HBM portion of the fusion not only bound heparin (as previously confirmed), but also presented heparin in a functional conformation, at least in the intrinsic and common coagulation pathways. Clotting was only slightly delayed (from 9 min to 13 min, Table 2) when SX2 was exposed to heparin, confirming that anticoagulant effects were primarily attributed to the addition of HBM and not the MaSp2 silk component. Regardless of the protein in each assay well, in the absence of heparin, clot formation occurred approximately 9 min after the addition of calcium chloride to the assay to chelate the sodium citrate anti-coagulant used during collection.

In addition to the ability of our new silk–HBM fusion construct to bind functional heparin, S4H4 was designed to act as an antimicrobial coating. The antimicrobial efficacy of the heparin-binding motif is based on its Antimicrobial Peptide (AMP)-like residues and structure. HBM includes a common motif component (XBBXBX, where X represents hydrophobic or uncharged amino acids, and B represents basic amino acids) that folds as an amphipathic helical structure with approximately 20Å between basic amino acids [68]. While recent evidence suggests a diversity of mechanisms based on the peptide sequence of the AMP but also AMP/bacterial pairings [69,70], the AMP structure remains important. The classic proposed mechanism of action suggests that AMP exerts bactericidal effects by disrupting the outer microbial member to create pores [69,71], without a specific target [68]. This action would seem to necessitate a soluble, amphoteric species, a supposition at odds with the proposed surface-bound use of the HBM silk fusion explored in this study. Nevertheless, based on a standard Kirby Bauer zone of inhibition assay (Figure 5) as well as a crystal violet biofilm assay (Figure 6), silk modified with HBM reduces the proliferation of both planktonic (Figure 5) and adherent (Figure 6) *E. coli 12435* bacteria. Thus, a more accurate predicted HBM mechanism of action may be based on the imperfect amphiphilic nature of the molecule, which may facilitate disruption of the bacterial membrane. Under this proposed mechanism, surface-bound HBM may prove more effective against sessile than planktonic pathogens. Confirmation of this mechanism requires further detailed structural and mechanistic studies with the silk fusion peptide.

The final consideration when interpreting the antimicrobial efficacy of S4H4 may be the length of the HBM element. The HBM chosen in this study is comprised of eight amino acids, with choice driven largely by the reported disassociation kinetics with heparin. However, it is unclear if a minimum peptide length is necessary for AMP activity. Liu et al. investigated the influence of AMP length on the hemolytic and antimicrobial activity of a model AMP, Arg-Trp (RW). The study found that (RW)_3_, a 6-amino acid peptide, was the optimal length for both efficiency in synthesis and antibacterial activity [72]. These results must be reconsidered when applying them to the current design, which includes a combination of Arg, Ala, and Lys. Inclusion of the aromatic amino acid Trp in the study by Liu and its absence in our study may significantly impact the relevance of the Liu analysis. Moreover, in the current AMP-like peptide, Ala and Arg, which can promote binding of heparin [73] but may prove otherwise disruptive when trying to preserve the mechanics of silk, need to be somewhat limited to balance the mechanics and desired anticoagulant function. Similarly, the presence of Pro in the MaSp2 silk portion of the fusion protein is generally considered disruptive to binding heparin, [27] but may play an important role in enhancing the efficacy of the AMP-like functionality [69], offering further constraints to the design.

The presence of Pro in MaSp2 is essential to maintain the balance of strength and elasticity [41]. Based on anticipated intermolecular hydrogen bonding [74], two polymerized MaSp2 silk monomers were predicted to provide a sufficient mechanical background to embed HBM and subsequently bind heparin with minimal steric hindrance. However, it is clear from the tensile testing (Figure 8) that the presence of HBM reduced the fiber strength while enhancing its extensibility. Although this may be attributed to the well-documented phenomena that larger diameter fibers are generally more extensible, the large deviations would negate this as being the sole cause of the effect. Instead, we would posit that the basic and cationic charges present in the HBM elements of S4H4 may disrupt intrinsic silk hydrogen bonding and hydrophobic interactions. Disruption of these critical forces may negatively affect the packing efficiency and alignment of the protein, effectively reducing the strength of the resulting fiber. Ultimately, having a heparin-binding, silk-based protein with increased extensibility and decreased tensile strength may prove beneficial as a biomaterial surface coating.

## 10. Conclusions

A new protein fusion comprising spider silk-derived MaSp2 peptides genetically linked with a consensus heparin-binding peptide was shown to bind heparin both at a surface and in buffer solution. This fusion protein was shown to exhibit affinity for complexing soluble heparin, demonstrated under certain in vitro conditions (e.g., ELISA and dot blot assay). However, the precise complex stoichiometry and heparin binding kinetics remain undetermined. Bound heparin anticoagulant activity was shown in an aPTT assay with rat plasma, an indirect coagulation in vitro assay. Furthermore the S4H4 fusion protein also demonstrated in vitro antimicrobial activity against both planktonic and adherent *E. coli ATCC 12435* cultures using zone of inhibition and crystal violet assays. This newly engineered chimera protein with its ability to bind heparin as well as its intrinsic antimicrobial potential can be utilized as a new biomedical material coating, potentially on hemodialysis catheters.

## Figures and Tables

**Table 1:** Amino acid sequences of the two expressed clones. Silk monomers are separated by lines while HBM component are shown in bold.

SX2	GGYGPGQQGPGGYGPGQQGPSGPGSAAAAAAAA|GGYGPGQQGPGGYGPGQQGPSGPGSAAAAAAAA
S4H4	GGYGPGQQGPGGYGPGQQGPSGPGSAAAAAAAA|GGYGPGQQGPGGYGPGQQGPSGPGSAAAAAAAA|GGYGPGQQGPGGYGPGQQGPSGPGSAAAAAAAA|GGYGPGQQGPGGYGPGQQGPSGPGSAAAAAAAA| **ARKKAAKA ARKKAAKA ARKKAAKA ARKKAAKA**

**Table 2:** A comparison of average times needed for visual clot formation either in the presence or absence of heparin.

Sample	Heparin Added	Clotting Time (min)
Heparin	+	No Clot
S4H4	+	No Clot
SX2	+	13 ± 5
Plasma only	+	30 ± 6
S4H4	-	9 ± 4
SX2	-	9 ± 3
Plasma only	-	8 ± 3

## Data Availability

Not Applicable.

## Supplementary Materials

The following supporting information can be downloaded at https://www.mdpi.com/article/10.3390/bioengineering9020046/s1; Figure S1: HPLC analysis of both the recombinant MaSp2 silk protein (flat grey line) and the s2H4 chimera protein (black peak). Each protein was derivatized with OPA to detect an increase in the number of primary amines present in the proteins. Notice the distinct peak at 32 min for the chimera that is absent in the silk protein, indicating an increase in primary amines as a result of the lysine residues in the HBM portion of the protein. Table S1: XPS analysis of the S_4_H_4_ coated wells after rinsing with the buffer showed an increase in the nitrogen content compared to the uncoated wells.

## Abbreviation

S4H4	fusion protein containing four repetitive units of silk and four repetitive units of heparin binding peptide
SX2	two repetitive units of silk
HBM	heparin binding motif
AMP	antimicrobial peptide
