# Supplementary material for: Anti-Coagulant and Antimicrobial Recombinant Heparin-Binding Major Ampullate Spidroin 2 (MaSp2) Silk Protein
Source: Bioengineering (Basel). 2022 Jan 19;9(2):46. doi: 10.3390/bioengineering9020046 (PMC8869596; doi:10.3390/bioengineering9020046)
Supplement: Supplementary file 1 [file bioengineering-09-00046-s001.zip › bioengineering-1512648-supplementary.pdf]

bioengineering

MDPI

Supplementary Information

Materials and Methods:

High Performance Liquid Chromatography (HPLC) – Purified proteins were dialyzed against water for 2 days using a 3 kDa-cutoff dialysis membrane and concentration was determined *via* absorbance at 280 nm as well as on SDS PAGE by comparison with BSA protein standards using optical densitometry. Subsequently, silk and silk-HBM recombinant proteins were derivatized with o-phthaldehyde (OPA, Sigma), that reacts with protein primary amines and fluoresces (ex = 360 nm, em = 460 nm) to allow detection. Briefly, 50 mg of OPA (powder, Sigma) was dissolved in 4 ml of methanol, 0.5 ml of potassium borate (0.5 M boric acid adjusted to pH of 10.4 with potassium hydroxide), and 50 µl 2-mercaptoethanol (Sigma) was added. This solution was kept at 4°C in the dark. Working reagent was prepared fresh by adding 50 µl OPA stock solution to 1 ml of 0.5 M potassium buffer. Each reaction contained 100 µl of protein solution in water, 100 µl of isopropanol and 200 µl of OPA reagent. Reactions were allowed to incubate at room temperature protected from light for 30 minutes prior to injecting on the HPLC column. Each reaction (10 µl) was injected on a C-18 Gold column with an inline guard column (ThermoFisher Scientific). A blend of acetonitrile and phosphate buffer (52:48) was used as the mobile phase (flow rate = 1 ml/min). Column temperature was set to 22°C.

X-ray Photoelectron Spectroscopy - X-ray Photoelectron Spectroscopy (K-Alpha™ X-ray Photoelectron Spectrometer, Thermo Scientific, MA) was used to analyze the elemental surface composition of the coated and uncoated surface. Survey spectra were obtained from -10 to 1350 eV to detect elements present on the surface with a pass energy of 200 eV and 1 eV energy step size. High resolution (HR) analyses with pass energy of 40 eV were performed at a take-off angle of 90° to determine elemental atomic percent composition. HR scans were performed at 50 eV pass energy for O 1s, C 1s, and N1s scans.

Results and Discussion

Since sizes of the MaSp2 silk alone versus that of the silk-HBM fusions were difficult to confidently distinguish by SDS-PAGE alone, each protein was derivatized with fluorescent tag, OPA, for detection with HPLC. OPA reagent derivatizes primary amines, producing a fluorogenic compound, which can then be detected. Pure MaSp2 silk protein is only expected to have a single free amine available for such derivatization on its N-terminus; whereas, the addition of the HBM peptide (ARKKAAKA) provides three lysine residues, equivalent to three additional primary amines (12 primary amines total) available for derivatization. Thus, HPLC was not used to detect a change in the molecular weight of the protein, but merely a change in the sequence of the protein as a confirmation that the HBM peptide was added. Pre-column OPA derivatization followed by separation with a C18 HPLC column provided separation of a new protein peak eluting at approximately 32 minutes (Supp Figure 1). The presence of this peak in an S_2H_4 sample is again likely the result of the increased number of primary amines corresponding to an increased molar percent of lysine in the S_2H_4 fusion. Unfortunately, degradation seemed to significantly impact the HBM fraction of the fusion as storing the protein at 4°C for 4 months caused the HPLC peak to not only broaden but also to shift to the left (i.e., retention time = 2-4 minutes), making it only slightly greater than the minimal peak detected for pure silk (data not shown). Replicate SDS-PAGE analysis run on these protein samples after storage validated the HPLC results, demonstrating not only a slightly higher concentration of silk but also the apparent degradation of the fusion protein, as indicated by a slight decrease in molecular weight (data not shown). Thus, this OPA fluorogenic detection method exploiting the increase in number of available primary amines for protein product derivatization allowed identification of the recombinant silk-HBM fusion protein. The XPS survey shows an increase in Nitrogen content for the coated surface due to the

presence of protein which confirms the adherence of the protein to the surface. It also shows a decrease in Si2p as the silicon surface is coated and covered with the protein (Supp Figure 2).

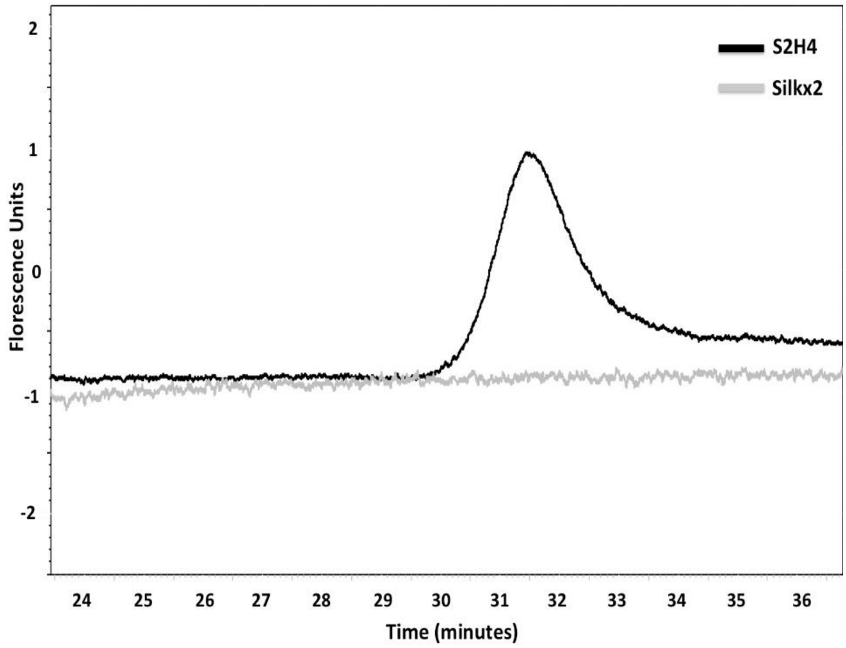

Figure S1. HPLC analysis of both the recombinant MaSp2 silk protein (flat grey line) and the s2H4 chimera protein (black peak). Each protein was derivatized with OPA to detect an increase in the number of primary amines present in the proteins. Notice the distinct peak at 32 minutes for the chimera that is absent in the silk protein, indicating an increase in primary amines as a result of the lysine residues in the HBM portion of the protein.

Table S1. XPS analysis of the S_4H_4 coated wells after rinsing with the buffer showed an increase in the nitrogen content compared to the uncoated wells.

Name	Coated %	Uncoated %
C1s	71.25	77.75
O1s	16.58	14.86
N1s	6.12	1.57
Cl2p3	3.4	3.18
Na1s	0.66	0.38
Si2p	0.91	2.26
